# New Materials for Orthodontic Interceptive Treatment in Primary to Late Mixed Dentition. A Retrospective Study Using Elastodontic Devices

**DOI:** 10.3390/ma14071695

**Published:** 2021-03-30

**Authors:** Grazia Fichera, Stefano Martina, Giuseppe Palazzo, Rosaria Musumeci, Rosalia Leonardi, Gaetano Isola, Antonino Lo Giudice

**Affiliations:** 1 Department of General Surgery and Surgical-Medical Specialties, School of Dentistry, University of Catania, Via S. Sofia 78, 95124 Catania, Italy; grazia.fichera@unict.it (G.F.); gpalazzo@unict.it (G.P.); rosariamusumeci@tiscali.it (R.M.); rleonard@unict.it (R.L.); nino.logiudice@gmail.com (A.L.G.); 2 Section of Orthodontics, Department of Biomedical and Dental Sciences and Morphofunctional Imaging, University of Messina, Via Consolare Valeria 1, 98123 Messina, Italy; 3 Department of Medicine, Surgery and Dentistry “Scuola Medica Salernitana”, University of Salerno via Allende, 84081 Baronissi, Italy; smartina@unisa.it

**Keywords:** orthodontic treatment, dental materials, elastodontic device, elastomeric appliance

## Abstract

The aim of this study was to assess the skeletal and dentoalveolar changes obtained after 1 year of treatment with elastodontic appliances (EA) in a retrospective cohort of children reporting early signs of malocclusion. Also, a detailed description of the tested EAs was reported. The study sample included 20 subjects, 8 males and 12 females, with a mean age of 8.4 ± 0.6 years, and a control group consisting of 20 subjects, 9 males and 11 females, with a mean age of 8.1 ± 0.8 years. All subjects in the treated group received the AMCOP second class (SC) (Ortho Protec, Bari, Italy) device. Digital impressions were taken along with a digital bite registration in centric relation before treatment (T0) and after 1 year (T1). Lateral cephalograms were also taken at T0 and T1 and cephalometric analysis was performed to assess the skeletal sagittal changes of the maxilla and the mandible (sella, nasion, A point angle, SNA^; sella, nasion, B point angle, SNB^; and A point–nasion–B point angle, ANB^) as well as the changes of the inter-incisors angle (IIA^). In the treated group, the distribution of subjects according to the presence of crowding and the pattern of malocclusion changed at T1. In the same group, there was an increase of subjects showing no signs of crowding and a class I occlusal relationship, while in the control group, there was a small increase of subjects developing dental crowding and featuring a worse sagittal relationship (class II) compared to pre-treatment condition. A statistically significant reduction of the overjet and overbite was recorded in the treated group between T0 and T1 (*p* < 0.05); in the control group, a slight increase in the overjet and overbite was detected at T1, being this increment significanct only for the latter parameter. In the tested group, no significant differences were found between SNA^ values detected at T0 and T1 (*p* > 0.05), instead the SNB^, ANB^, and IIA^ showed a significant increase after 1 year of treatment (*p* < 0.05). From a clinical perspective, all clinical goals were reached since patients showed remarkable improvements in overjet, overbite, crowding, and the sagittal molar relationship. Within the limitations of the present study, EAs could be effectively used for the interceptive orthodontic in growing patients.

## 1. Introduction

Interceptive orthodontic therapy represents a preventive approach for treating malocclusion at pediatric age. It is based on the rationale that signs of different malocclusions are often detectable in early to late mixed dentition [1,2] and that many forms of malocclusions, in general, are not self-correcting with age [3]. However, a consensus of the effectiveness of interceptive therapy has not been reached since some studies suggest that early phase treatment might determine a stable post-treatment occlusion [4,5], while other studies confirm that children would not benefit from early treatment apart from a transient increase in self-esteem as compared with late treatment in one phase [6].

Elastodontics is an interceptive therapy that uses removable appliances made with silicone elastomer to produce light and biological elastic forces to correct malocclusions, correcting the position of the teeth and potentially affecting growth [7,8]. In this regard, they have been designed mainly for the treatment of orthopedic—orthodontic problems of the evolutionary age and, therefore, are used in deciduous or mixed dentition [9], with the assumption of improving sagittal and vertical relations and the incisors’ alignment at the same time. 

The first elastomeric appliance (EA) was the Occlus-O-Guide^®^ (Ortho-tain, Winnetka, IL, USA), also known as the Eruption Guidance Appliance (EGA), designed by Bergersen. This appliance features two double-matched planes, upper and lower, that guide the position of the teeth in the dental arches. The elastomeric material allows the tooth movement to occur in synergy with the neuromyofascial system and function; moreover, the presence of specific flanges prevent the perioral muscles from affecting on tooth movement [10]. For these characteristics, this appliance is generally used in the finishing stage of the orthodontic treatment or before starting orthodontic treatment with the aim of managing minor tooth movement correction [11]. 

WIth the employment of the Occlus-O-Guide or similar devices in daily practice, the number of elastomeric preformed appliances has grown considerably [12,13]. In this regard, different appliances have been designed with the aim of extending their function to the correction of skeletal and vertical disharmonies between the maxilla and the mandible.

As functional appliances, they promote mandibular advancement to correct class II discrepancies and feature a vertical opening in the anterior region to provide a greater vertical development of the posterior teeth. As positioners, they allow minor tooth movement or they guide the eruption of the anterior teeth as a result of the elastomeric material. In this regard, the employed materials are soft enough to allow patient compliance without traumatizing the oral mucosa and jaws, which is of great clinical relevance considering that patients’ compliance represent on of the main issues of the orthodontic treatment in young subjects [14,15].

There is a complete array of activators for every type of mouth, according to the skull conformation, body features, and dental arch shape. They are designed for arches with deciduous dentition, mixed and permanent, and are therefore of increasing size. The main clinical applications of elastodontic appliances are: increased overjet and overbite, gummy smile, anterior crowding and rotations, open bite, class II malocclusion, and scissors-bite.

However, despite the increasing usage of EAs for treating malocclusion at young age, the literature is lacking studies assessing their potential effectiveness. In this regard, this retrospective study aimed to evaluate the skeletal and dentoalveolar changes obtained after 1 year of treatment with an EA in a cohort of subjects with a class II skeletal pattern associated with other clinical signs of malocclusions and to discuss the potential benefits and disadvantages of interceptive therapy with these appliances.

## 2. Materials and Methods

### 2.1. Study Sample

For the purpose of the present study, subjects were recruited from a retrospective cohort of patients treated at the Department of Orthodontics, University of Catania, Italy, in accordance with the regional health protocol for assuring orthodontic treatment to patients with limited or poor economic resources. Subjects were included according to the following criteria: age between 6 and 11 years, mixed deciduous dentition with upper central incisors and first molars fully erupted, radiographic and photographic records taken before treatment (T0) and at one-year follow-up (T1), skeletal and/or dental class II malocclusion, overjet ≥ 4 mm, overbite ≥ 2/3 mm, or anterior crowding in combination with an overjet of ≥ 4 mm. Exclusion criteria were: previous orthodontic therapy, class III malocclusion and retroclined upper incisors, systemic disease, or signs of temporomandibular dysfunction.

A control group of subjects featuring the same inclusion/exclusion criteria, and that could not adhere to the regional protocol, was also recruited. The treatment group consisted of 20 subjects, 8 males and 12 females, with a mean age of 8.4 ± 0.6 years, while the control group consisted of 20 subjects, 9 males and 11 females, with a mean age of 8.1 ± 0.8 years.

### 2.2. Treatment Protocol

**Figure 1:**
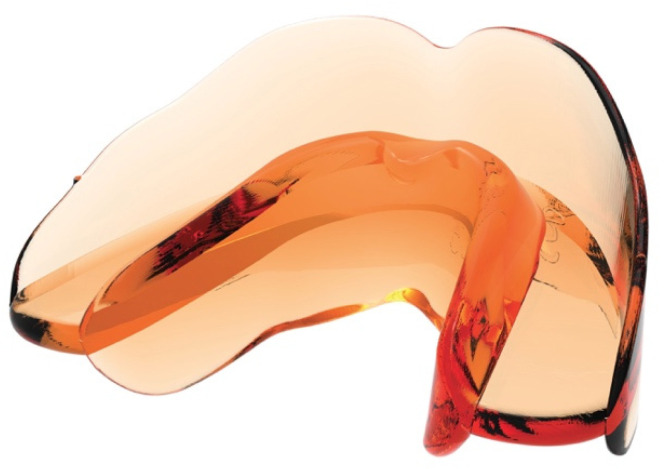
AMCOP second class (SC) appliance.

All subjects in the treated group received the AMCOP second class SC (Ortho Protec, Bari, Italy). Briefly, AMCOP second class SC is a preformed elastomeric device that features two flanges, one on the vestibular side and one on the lingual side, with a free central area in which the teeth can be positioned without any constraints. These double flanges are linked by an occlusal guide (anterior slope) that keeps the two arches in normo-occlusion, simulating a class I relationship by leading the mandible in a forward position. Figure 1 shows an example of the AMCOP second class SC appliance used in the cohort of subjects of the present study.

The device is made of a polymer/elastomer combination. The material is very elastic, soft, comfortable, and non-deformable. In addition, it is thermoactive, and adaptable to different arch shapes. Possible interference from flanges can be modified with heat-appropriate instruments. The patient can also expand the device by soaking it in hot water at about 70 °C for 30 s. To fix it in its new form, it can be soaked in cold water. 

The device does not feature indentations, avoiding teeth constriction or the generation of orthodontic movement; instead, it simply favors the establishment of a normal eruption pattern along with a harmonic growth of the dentoalveolar structures in all three dimensions. This device is designed with a mandibular sliding plane that places the incisors head to head causing a protrusive posture of the mandibular during wearing. It also allows the placing of the tongue in the correct posture at the palatine spot, eliminating any interference of the tongue and of dysfunctional orbicular muscles on the teeth. Since the appliance is preformed, the appropriate size was selected for each patient according to the molar diameter and the incisors’ inclination. All subjects were asked to wear the device at night and for an hour during the day. During daylight hours, the patient had to bite the device while keeping their lips in contact.

Digital impressions (Carestream 3600, Carestream Dental LLC, Atlanta, GA, USA) were taken along with a digital bite registration in centric relation before treatment (T0) and after 1 year (T1). Lateral cephalograms (ORTHOPHOS XG, Sirona Dental GmbH, Wals bei Salzburg, Austria) were also taken at T0 and T1. Children in the control group were offered the opportunity to receive the treatment one year later. During this period, they were monitored and underwent digital impressions, as the treated subjects; however, no radiographic examination was taken to avoid unnecessary radiation because of ethical restrictions [16].

### 2.3. Data Measurements

All digital dental models were imported into Ortho Analyzer software (3Shape A/S, Copenhagen, Denmark) in order to evaluate the dentoalveolar components of the malocclusion. In particular, the following parameters were recorded both at T0 and T1:

- Overjet (mm).
- Overbite (mm).
- Crowding (distribution): (1) aligned dentition, (2) mild crowding (≤2 mm), (3) moderate crowding (3–4 mm).
- Severe crowding (>4 mm).
- Angle malocclusion (distribution): class I, class II, class I/II.

**Figure 2:**
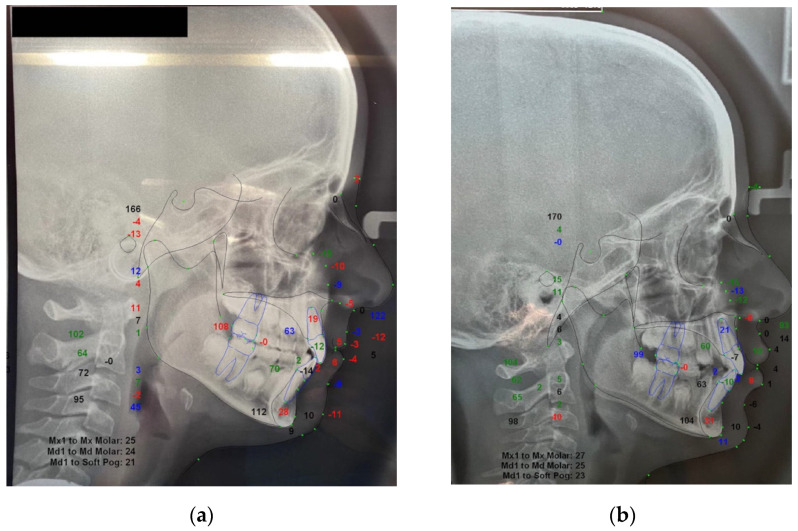
Comparative evaluation of cephalometric analysis performed at T0 (**a**) and T1 (**b**) to identify potential skeletal and dentoalveolar changes in sagittal and vertical relation.

Cephalometric analysis was also performed at T0 and T1 for the treatment group by using the Dolphin3D software (Dolphin Imaging, version 11.0, Chatsworth, CA, USA), and the following parameters were recorded for a descriptive evaluation of the clinical outcomes: SNA^, SNB^, ANB^, and interincisal angle (IIA^) (Figure 2). Both measurements performed on digital study models and cephalograms were performed by the same expert operator (A.L.G.) In this regard, all digital models and cephalograms were blinded by using specific labels in order to hide any identification of the included subjects.

### 2.4. Statistical Analysis

The Shapiro–Wilk test and Levene’s test were used for the assessment of data distribution and equality of variance. Since data showed a normal data distribution, parametric tests were used. In particular, paired Student’s *t*-test and chi-square test were used to compare data obtained at T0 and T1 in the control group, and an unpaired Student’s *t*-test was used to compare data changes obtained between groups (only data from the analysis of study models). Statistical significance was set at *p* < 0.05. Ten patients were randomly selected, and the entire procedure was repeated by the same expert investigator after 4 weeks. Intra-examiner reliability was assessed using an intraclass correlation coefficient (ICC). Data sets were analyzed using SPSS^®^ version 24 Statistics software (IBM Corporation, 1 New Orchard Road, Armonk, NY, USA).

## 3. Results

Concerning the measurements performed on digital study models, a statistically significant reduction of the overjet and overbite was recorded in the treated group between T0 and T1 (*p* < 0.05); in the control group, a slight increase in the overjet and overbite was detected at T1; however, significance was found only for the latter parameter (Table 1).

In the treated group, the distribution of subjects according to the presence of crowding and the pattern of malocclusion changed at T1. In particular, there was an increase in subjects showing no signs of crowding and class I occlusal relationship; however, in the control group, there was a small increase in subjects developing dental crowding with a worsening of the sagittal relationship (class II). However, no significant differences were found between the two groups both at T0 and T1 (Table 2 and Table 3).

Concerning cephalometric measurements, the tested group showed no significant differences between SNA^ values detected at T0 and T1 (*p* > 0.05), however, SNB^, ANB^, and IIA^ showed a significant increase after 1 year of treatment (*p* < 0.05) (Table 4).

Concerning the reliability of the methodology, no differences were found between intraoperator readings, with excellent correlation indexes ranging from 0.915 to 0.934 for measurements performed on digital models and ranging from 0.921 to 0.944 for cephalometric measurements.

## 4. Discussion

The present study assessed the potential effectiveness of interceptive orthodontic treatment with an elastodontic appliance in subjects with specific early signs of malocclusion (mixed dentition stage).

According to our findings, all subjects treated with elastodontic appliance (EA), showed a significant improvement of the overjet, overbite, crowding, and sagittal molar relationship. Both skeletal and dentoalveolar changes have contributed to the resolution of the malocclusion, suggesting that elastodontic appliances may represent a comprehensive early treatment method. These findings may support previous evidence showing that one phase of treatment with elastodontic devices followed by a long retention period, including adolescence, might be an effective and alternative approach to the conventional biphasic protocol with a functional device and further treatment with a fixed appliance [17,18].

Concerning dentoalveolar effects, all patients showed a controlled eruption of the maxillary incisors inhibiting further overeruption, with the concomitant eruption of the posterior teeth These effects are induced by the specific design of the AMCOP device, which is thicker in the anterior section and remarkably thinner in the posterior section, providing differential control for the vertical pattern of the eruption. In the lower arch, the same mechanism, in combination with the anterior sloped plane for mandibular advancement, generates a force-system that induces vertical control associated with protrusion of the incisors and a mesial advance of the molars. The combined effects on the two arches produces, in class II subjects, the correction of the molar relationship, and the reduction of the overjet with the improvement of the overbite [19].

The modification of the dentoalveolar components induced by EAs has been documented with data from cephalometric analysis and assessment of gypsum models [13]. Furthermore, the correction of the overjet and overbite would increase the stability of normal vertical incisor relationships preventing or mitigating the post-treatment overeruption [8,17,20]. In this regard, according to previous evidence, it would seem appropriate to start treatment slightly before the eruption of the incisors since, at this stage, the EA acts as an erupting guide that favors 1) the establishment of normal contact with adequate torque of the incisors, 2) a significant improvement of anterior crowding supported by natural transversal growth of the dentoalveolar process [21].

Subjects in the treated group showed an increase of the mandibular sagittal projection, with a mean difference of 2.7° for SNB^ and 2° for ANB^. Thus, it may be postulated that the EA might stimulate mandibular growth even in mixed dentition, as also suggested by Keski-Nisula et al. [2,18]. In their study, they analyzed the cephalograms of 219 children, with 115 subjects out of the total sample being treated with an elastomeric appliance for an average period of 3 years. The average age of starting therapy was about 5 years. The difference between the initial and final mandibular length in the treated group was 11.1 mm compared to the 7.2 mm of the control group. Similarly, Janson et al. [19] analyzed the cephalograms of 60 patients with an average age of 9 years, half of whom were treated with EAs and the rest serving as controls. The authors found that, after 26 months, the difference in mandibular length was respectively 6.42 mm in the treated group and 3.87 mm in the control group. The different ages of the sample and the different duration of treatment may explain the discrepancy between the values found in the two studies [18,19]. None of the above mentioned researches showed skeletal effects on the upper jaw, in line with other studies of different functional equipment for the correction of skeletal class II. Accordingly, cephalometric assessment of the treated subjects did not show relevant changes to the maxillary bone that may have contributed to the resolution of class II malocclusion [22]. Concerning vertical skeletal components, four patients treated with the EAs in the present study showed an increase in anterior vertical dimension, which corroborates previous data but does not agree with other evidence [22,23].

An interesting innovation of elastodontic appliances lies in the soft and elastic material, which allows the possibility of performing myofunctional exercises to rebalance the oral and lingual musculature. Most importantly, the improvements in both skeletal and dentoalveolar components of the malocclusion may be responsible for the restoration of normal muscular activities. In this regard, previous evidence from electromyography (EMG) studies would suggest that one of the most frequently observed effects of wearing a myofunctional appliance is the reduction of the hyper-tone of the mental muscle and an increase in muscle activity of the orbicular, usually hypotonic in cases of labial incompetence [12,24]. Moreover, since elastomeric equipment acts as a shield that isolates the dentoalveolar structures from the perioral muscles, previous evidence would suggest that it is possible to achieve the rebalancing of the perioral musculature similar to that obtainable with rigid functional equipment, such as the Fränkel appliance [25]. Most of the beneficial effects of the treatment with EAs are consistent with those reported with the use of functional appliances, such as Fränkel, Twin block, and other appliances [24,26]. In this regard, it was recently found that the continuous and correct use of the functional device induced measurable intraoral (dental arches) and extraoral (face) morphological modifications [24,27].

The market of EAs is continuously growing and offers a plethora of devices; however, this is in contrast with the lack of adequate scientific evidence validating their clinical usage. It should be noted that all of the cited studies, except for one [16], consist of a retrospective cohort of studies due to the difficulties in designing studies involving the treatment of growing subjects. Prospective randomized clinical studies are warmly recommended to investigate the effectiveness of EAs in comparison with control groups and the functional appliance therapies that, instated, are widely supported by scientific evidence. 

**Figure 3:**
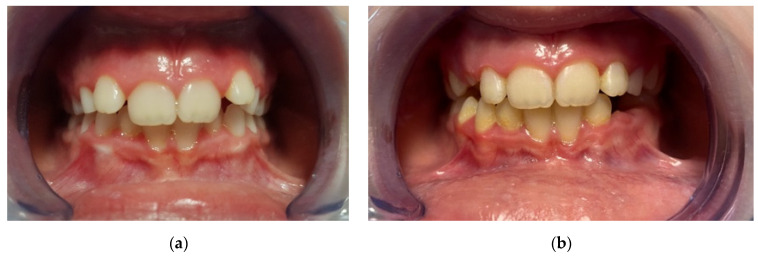
Intraoral frontal photographs of one of the included subjects treated with AMCOP device. (**a**) pre-treatment (T0), (**b**) post-treatment (T1).

All subjects presented have reported excellent compliance with the AMCOP device. A common issue was excessive salivation, this effect, however, gradually decreased after a few days. One of the potential advantages of an EA over functional appliances is that they are well accepted by children since they do not require dental impressions, and they are asked to wear the appliance only at night and for a few hours in the afternoon. Furthermore, due to its lower cost compared to treatment with a functional appliance, interceptive therapy with an EA could be an important alternative for treating subjects with difficult financial conditions (Figure 3).

### Limitations

The retrospective design, and the small sample size represent the main limitations of this study. In this regard, long-term clinical trials with a large sample size and parallel arms are warmly recommended to investigate the effectiveness of interceptive orthodontic treatment in subjects with different skeletal and dentoalveolar characteristics and to validate the routine use of elastodontic appliances under specific circumstances in daily clinical practice.

## 5. Conclusions

According to the findings of the present study, all of the subjects treated with an elastodontic appliance showed a significant improvement of specific clinical signs of malocclusion, such as overjet, overbite, crowding, and the sagittal molar relationship. As a consequence, EAs may present a simple, natural, and less invasive therapeutic option for treating malocclusion under specific circumstances. Furthermore, they may be considered a valid therapeutic device for assuring orthodontic treatment of patients with limited or poor economic resources.

## Figures and Tables

**Table 1:** Inferential statistics of overjet and overbite detected within treated and control groups. T0 = pre-treatment, T1 = post-treatment, SD = standard deviation * = significance set at *p* < 0.05 and based on paired Student’s *t*-test for inter-timing comparisons; ** = significance set at *p* < 0.05 and based on independent Student’s *t*-test for intra-timing comparisons.

Parameters	Treated Group					Control Group					Significance **
	T0		T1		Significance *	T0		T1		Significance *	
	Mean	SD	Mean	SD		Mean	SD	Mean	SD		
**Overjet**	5.1	0.8	2.5	0.5	*p* < 0.05	4.7	1.1	5	1.2	NS	*p* < 0.05
**Overbite**	4.5	0.9	1.9	1.2	*p* < 0.05	4.2	1.3	4.9	1.4	*p* < 0.05	*p* < 0.05

**Table 2:** Inferential statistics of data distribution of crowding between treatment group (TG) and control group. T0 = pre-treatment, T1 = post-treatment, SD = standard deviation, *n* = subjects’ number; significance set at *p* < 0.05 and based on chi-square test.

Parameters	T0			Significance	T1		
	Subjects	TG	CG		TG	CG	Significance
		*n*	*n*		*n*	Mean	
**Maxilla**	**Crowding**	14	11	NS	10	15	NS
	**Normal**	6	9		10	5	
	**Total**	20	20		20	20	
**Mandibular**	**Crowding**	16	17	NS	6	20	NS
	**Normal**	4	3		14	0	
	**Total**	20	20		20	20	

**Table 3:** Inferential statistics of data distribution of Angle malocclusion between the treatment group (TG) and control group. T0 = pre-treatment, T1 = post-treatment, SD = standard deviation, *n* = subjects’ number; significance set at *p* < 0.05 and based on chi-square test.

Parameters	T0			T1		
	TG	CG	Significance	TG	CG	Significance
	*n*	*n*		*n*	Mean	
**Class I**	6	7	NS	13	8	NS
**Class II**	13	10		2	9	
**Class I/II**	4	3		5	3	
**Total**	20	20		20	20	

**Table 4:** Inferential statistics of overjet and overbite detected within treated and control groups. T0 = pre-treatment, T1 = post-treatment, SD = standard deviation; * = significance set at *p* < 0.05 and based on paired Student’s *t*-test for inter-timing comparisons.

Parameters	Treated Group				
	T0		T1		Significance *
	Mean	SD	Mean	SD	
**SNA^**	79.4	0.96	80.06	1.19	NS
**SNB^**	74.6	1.07	77.3	1.31	*p* < 0.05
**ANB^**	4.7	0.66	2.76	0.69	*p* < 0.05
**IIA^**	130.1	6.5	131.8	5.6	*p* < 0.05

## Data Availability

Data are available upon request.
